# Exploring the relationship between established HIV risk factors and depressive symptoms amongst young women without HIV in two sites in South Africa

**DOI:** 10.1371/journal.pone.0317732

**Published:** 2025-01-29

**Authors:** Zanenhlanhla Gumbi, Celia Mehou-Loko, Lindi Masson, Makhosazana Mdladla, Nokuthula Maphumulo, Jo-Ann Passmore, Sanele Mbeje, Linda Gail Bekker, Disebo Potloane, Heather Jaspan, Nina Radzey, Andrea Abrahams, Rushil Harryparsad, Pamela Mkhize, Hilton Humphries

**Affiliations:** 1 Centre for the AIDS Programme of Research in South Africa, Durban, South Africa; 2 Department of Biochemistry, School of Life Sciences, University of KwaZulu-Natal Pietermaritzburg, Pietermaritzburg, South Africa; 3 Department of Pathology, Division of Medical Virology, Institute of Infectious Disease and Molecular Medicine (IDM), University of Cape Town, Cape Town, South Africa; 4 Disease Elimination Program, Life Sciences Discipline, Burnet Institute, Melbourne, Australia; 5 Central Clinical School, Monash University, Melbourne, Australia; 6 National Health Laboratory Service (NHLS), Cape Town, South Africa; 7 Desmond Tutu Health Foundation, University of Cape Town, Cape Town, South Africa; 8 Seattle Childrens’ Research Institute and University of Washington, Seattle, Washington, United States of America; 9 Department of Paediatrics and Department of Global Health, University of Washington, Seattle, Washington, United States of America; 10 Centre for Community-Based Research, Human Science Research Council, Pretoria, South Africa; 11 Department of Psychology, School of Applied Human Sciences, University of KwaZulu-Natal, Durban, South Africa; 12 Division of Social and Behavioural Sciences, School of Public Health, Faculty of Health Sciences, University of Cape Town (UCT), Cape Town, South Africa; University of the Witwatersrand, SOUTH AFRICA

## Abstract

**Purpose:**

Adolescent girls are at high risk for depression and human immunodeficiency virus (HIV) acquisition. Poor mental health can increase vulnerability to risky sexual behaviours. Therefore, this study aims to determine the prevalence of depressive symptomology and explore the convergence of HIV risk factors with depressive symptoms amongst cis-gender adolescent girls and young women (AGYW) in rural KwaZulu-Natal (KZN) and peri-urban Western Cape (WC) communities in South Africa.

**Methods:**

Cross-sectional survey data from two sites in South Africa was used - the rural Vulindlela community in KZN and the peri-urban Philippi East community in the WC. Study inclusion criteria included being sexually active with at least one male partner, and not planning to relocate in the next 12 months. The PHQ-9 scale was used to determine depressive symptomology, a socio-behavioural questionnaire was used to determine sexual behaviours, odds ratios and confidence intervals derived from logistic regression models were used to explore the associations between depressive symptomology and socio-behavioural factors associated with HIV acquisition.

**Results:**

The cohort consisted of 274 adolescent girls, 38.6% from the WC site and 61.4% from the KZN site. Overall, 15.7% (43/274) of AGYW reported depressive symptoms. Participants from the peri-urban WC site were more likely to experience depressive symptoms (OR 8.34; 95% CI 3.80–18.30) compared to those living in the rural KZN site. Depressive symptoms were less likely to occur in adolescent girls between the ages of 14 to 17 as compared to those between the ages of 18 and 19 (OR 0.44; 95% CI 0.22–0.90). Socio-behavioural HIV risk factors associated with depressive symptoms include: age disparate relationships (OR 2.98; 95% CI 1.52–5.84), high (four or more) numbers of lifetime partners (OR 8.15; 95% CI 3.60–18.45) and engaging in sex under the influence of alcohol (OR 2.58; 95% CI 1.32–5.04). Multivariate analysis showed that participants from the WC site (AOR 5.25; 95% CI 1.95–14.17) had higher odds of experiencing depressive symptoms while participants with four or more lifetime partners (AOR 3.46; 95% CI 1.24–9.60) were at higher odds of experiencing depressive symptoms.

**Conclusion:**

In this cross-sectional study, depressive symptomology is associated with certain HIV risk behaviours. Longitudinal studies are required to test the causal relationship between depression and HIV acquisition and to better understand the geospatial differences observed.

## Introduction

Progress has been made in reducing new HIV infections with a notable 60% decrease observed globally HIV in 2023. However, women and girls represented 44% of all new infections during this period [1]. HIV still continues to be a significant factor contributing to poor health outcomes, particularly for Adolescent Girls and Young Women (AGYW) living in sub-Saharan Africa (SSA). Among those who acquired HIV in 2023, 62% were AGYW aged 15–24 years in SSA [1]. Moreover, AGYW were three times more likely to contract HIV compared to their male peers among adolescents with new infections. Mental health is acritical mediator of poor HIV treatment outcomes and may also increase the risk of HIV acquisition. Understanding the impact of mental health issues on HIV acquisition risk in endemic regions is essential for developing more effective interventions.

Depression - a depressed mood or loss of pleasure, or interest in activities for long periods [2] - is the most prevalent mental health disorder globally [3,4]. There are an estimated 280 million cases of depression worldwide [2], with ~ 80% of these occurring in low-and middle-income (LMIC) countries [4]. Similar to trends in HIV, young women aged 14–25 years are particularly vulnerable to depression, being three times more likely to report depression than their male peers [5]. In South Africa, data from a large national survey conducted in 2014 reported that one in four individuals experienced depression, with heterogeneity in prevalence across genders [6].

Clear evidence demonstrates a link between depression and HIV in sub-Saharan Africa. Depression negatively influences treatment adherence and mortality amongst people living with HIV, and an HIV diagnosis may contribute to experiencing depression as it is a life-threatening condition, is stigmatised and may have serious negative social consequences. Depression’s role in enhancing HIV acquisition remains an area requiring more research. Data from a randomised control trial conducted in Mpumalanga, South Africa suggests that socio-behavioural mediators explained two-thirds (indirect effect: OR = 2.4, 95% CI: 0.2–4.5) of the association between depressive symptoms and HIV incidence [7]. In AGYW living in Kenya, moderate-to-severe depression was associated with higher odds of being in age disparate relationships, engaging in transactional or forced sex, experiencing intimate partner violence and higher alcohol use, all of which are also well described risk factors for HIV infection [8]. Additionally, economic stress, lack of family support, substance use, unemployment, food insecurity, alcohol use, intimate partner violence, and childhood traumas have been shown to enhance depressive symptomology and are also risk factors for HIV infection [9–13].

There is a lack of evidence on depression amongst AGYW in general in South Africa, and in particular amongst rural AGYW. Understanding the association between depressive symptomology and HIV risk behaviours, and how this differs for AGYW living in urban and rural areas provides novel insight into this relationship and could inform future intervention development. The present analysis aimed to investigate the relationship between depressive symptoms (measured using the PHQ-9) and HIV risk behaviour among AGYW in two different HIV-endemic locations in South Africa.

## Methods

### Study setting

The Centre for the AIDS Programme of Research in South Africa (CAPRISA) site is based in the rural Vulindlela area in the uMgungundlovu district of KwaZulu-Natal (KZN), South Africa. The area is predominantly isiZulu speaking marked by high levels of poverty, poor infrastructure and high levels of sexually transmitted infections (STI) and HIV prevalence [14]. The Desmond Tutu Health Foundation site is located in the Mitchell’s Plain township area in Cape Town, Western Cape (WC), South Africa. Participants in that area face high levels of gangsterism, crime and violence, substance abuse, teenage pregnancy, and high levels of STI, HIV and tuberculosis [15].

### Cohort description

This study was nested within a longitudinal cohort study called MIST/MISC (Mucosal Injury from Sexual Trauma/Contact). MIST was a prospective, longitudinal observational study that enrolled adolescent girls (14–19 years) and adult women (25–35 years) at two sites in South Africa: 1) a rural site based at the Vulindlela Clinical Research (VRC) site at CAPRISA in Mafakathini, KZN from the 1^st^ of June 2018 until the 30^th^ of June 2021, and 2) a peri-urban site based within the Desmond Tutu Health Foundation (DTHF) Adolescent Clinic situated in Philippi East, WC from 1^st^ of July 2019 until the 30^th^ of November 2021. The MIST study aimed to investigate unique socio-behavioural, reproductive tract anatomical and biological characteristics around sexual debut in adolescent females (14–19 years) in response to early sexual exposure, male semen, vaginal products, mucosal trauma and wound healing compared to a group of older women (25–35 years). Both sites recruited study participants by mobilising existing networks and general community outreach activities. Eligibility criteria at both sites included being sexually active with at least one male partner to meet the aims of the parent study, being willing to provide contact information and not planning on moving out of the area in the next 12 months. Participants in the study were screened for pregnancy using urine pregnancy tests and for HIV using Uni-Gold™ Recombigen® HIV-1/2 (Trinity Biotech, Ireland) and Determine™ HIV-1/2 test (Abbott Diagnostics Scarborough, Inc., USA) in KZN. In WC, they were tested for HIV using One Step Anti-HIV (1&2) (Intec Products Inc., China), and HIV 1 + 2 Rapid Test (Wantai BioPharm, China). Participants were excluded if they were pregnant (due to extensive genital specimen sampling) or if they tested positive for HIV. They were then referred for appropriate antenatal care and/or HIV management. Participants aged 16 years and older provided written informed consent. The ethics committees in KZN and WC waived the requirement for parental consent for 16–17-year-olds due to the sensitive nature of the study. For girls aged 14–15 years, both assent and parental consent were obtained. Both study sites received ethical approval from their respective ethics committees, the Biomedical Research Ethics Committee at the University of KwaZulu Natal (UKZN) (BF504/17) and the Human Research Ethics Committee at the University of Cape Town (UCT) (696/2017).

### Study tools

At enrolment, a socio-behavioural questionnaire was completed at both study sites by participants with the assistance of trained nurses. Socio-behavioural variables were selected for inclusion based on a review of previous literature about factors that may be associated with increased HIV vulnerability and depression as well as factors hypothesized to be linked to depression and HIV in the South African context, including: (1) *socio-demographic factors:* age, location, education, employment status, financial support, and death in the household; (2) *sexual history and behaviour:* consent and age at sexual debut, contraception use, number of lifetime partners, partner age gap (a peer partner can be defined as a partner who is ≤ 5 years younger than participant, and a non-peer is ≥ 5 years older than participant), condom use at last sex act, use of sexual enhancers, engagement in transactional sex or sex under the influence of alcohol, and pregnancy history; and (3) *diagnosed biological factors and self-reported substance use:* body mass index (BMI), Chlamydia trachomatis (CT), Neisseria gonorrhoeae (NG), Trichomonas vaginalis (TV), bacterial vaginosis (BV), and substance use ever. STI testing was done using the Xpert® CT/NG assay (Cepheid, USA; KZN site), OSOM® *Trichomonas* Rapid Test (Sekisui Diagnostics, USA; KZN site) or genesig® kits (Primerdesign™ Ltd, UK; WC site). BV was diagnosed using Nugent scoring.

A patient health questionnaire (PHQ)-9 was completed by all women with assistance from a trained lay counsellor. The PHQ-9 is a depressive symptom screening tool that scores each of the nine DSM-IV (Diagnostic and Statistical Manual of Mental Disorders) criteria from “0” meaning not at all, to “3” meaning nearly every day [16]. This depression screening tool has been validated in South Africa [17]. The sum of each participant’s reported symptoms is then calculated and classified as being mild (scores of 5–9 out of 27), moderate (scores of 10–14 out of 27), moderately severe (scores of 15–19 out of 27), or severe (scores of 20–27 out of 27) [16]. For the current analysis, we considered a participant to exhibit depressive symptomology if she scored ≥ 5. Those who scored in the categories of mild to severe depressive symptomology received in-house counselling from qualified lay counsellors and were provided with a referral to a local social worker, helpline, or relevant local mental health services. In addition, those responding affirmatively to a question about contemplating self-harm or expressing suicidal ideation were immediately referred for assistance, and a facilitated referral to specialist services was completed.

The study teams translated both the socio-behavioural and PHQ-9 questionnaires from English to isiZulu and isiXhosa, then back-translated them into English to ensure reliability. Participants could choose the language of the questionnaire they were most comfortable with; isiZulu in KwaZulu Natal, isiXhosa in Cape Town, or English at both sites.

### Statistical analysis

The associations between sociodemographic characteristics, sexual behaviour, health-related behaviours, and substance use of participants with depressive symptoms, defined by a PHQ-9 score of ≥ 5, were compared to participants without depressive symptoms, defined by a PHQ-9 score of ≤ 4 using logistic regression. The logistic regression model has become the primary approach for examining relationships between binary outcomes and a set of exposure variables, and it offers the advantage of controlling for multiple confounders [18,19]. A univariable regression was performed first to assess whether there is an association between the outcome and each predictor. Multivariable regression was then used to investigate the association between depressive symptomology and a combination of several variables selected based on the literature and those found to be significant in the univariable regression (level of significance = p ≥ 0.05).

The association is described using unadjusted and adjusted odds ratios (AOR) with 95% confidence interval (CI) to provide evidence of precision. An OR of 1 or a CI that includes 1 indicates no association between the predictor and depression. The total number of participants included in the logistic regression model (univariable and multivariable) varied depending on the completeness of the predictor variables. Missing values were excluded from the regression model, the proportion of missingness was low ranging from 0% to approximately 6% except for miscarriage status (15.7%). The proportion of missing values in the multivariable model was 5.5%. We assessed the goodness of fit on the final logistic regression model using the Hosmer-Lemeshow test. All statistical analysis was done using SAS, version 9.4 [20].

## Results

### Demographic characteristics of the study cohort

**Fig 1:**
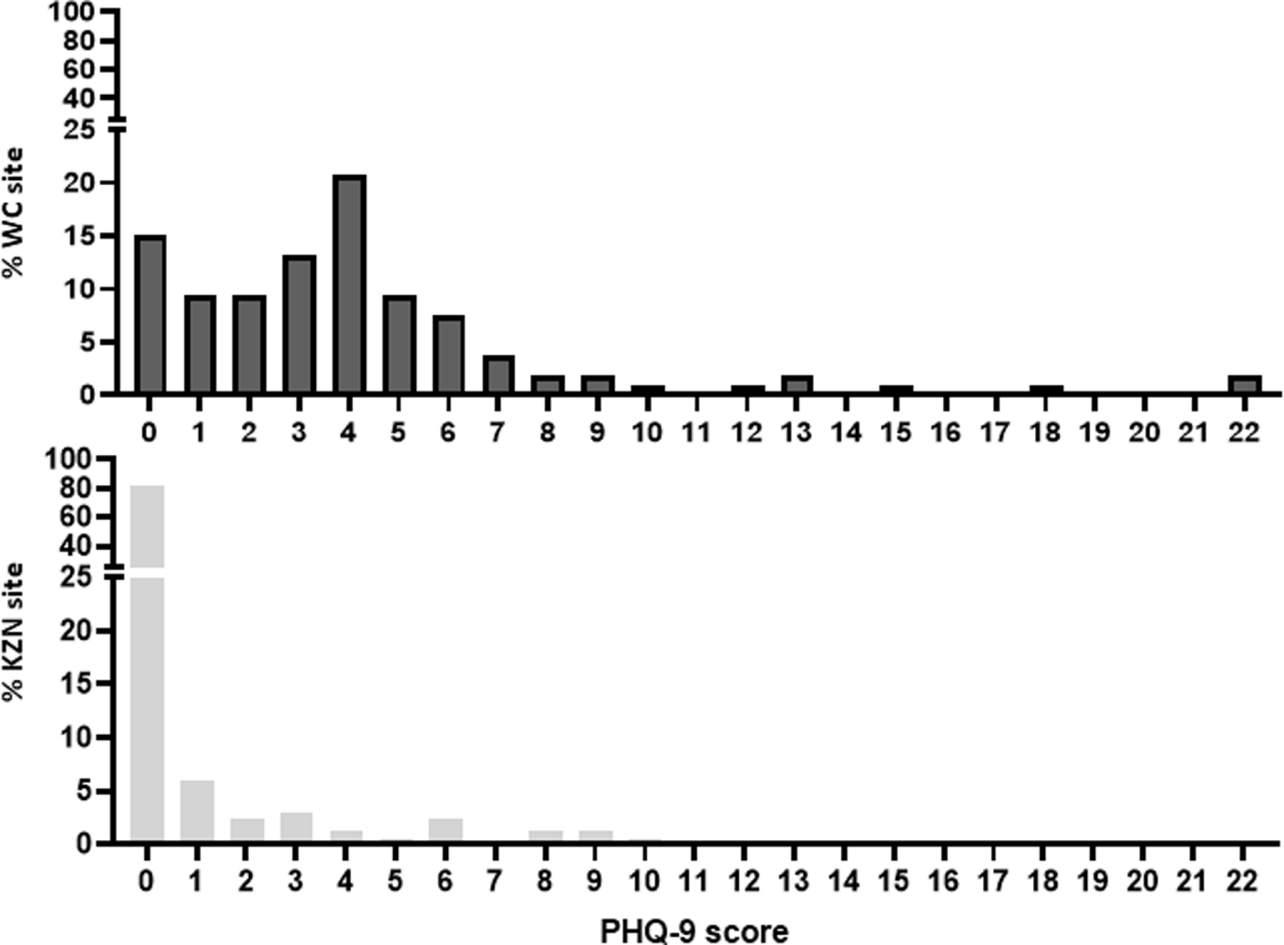
Distribution of participant depression PHQ-9 scores across Western Cape (WC) site and KwaZulu-Natal (KZN) site locations.

Of the 274 sexually active AGYW participants in this study, 106/274 (38.6%) were from the WC site and 168/274 (61.4%) were from the KZN site (Table 1 and S1 Fig Consort diagram). Based on the PHQ-9 assessment, 15.7% of the participants were classified as experiencing depression. The majority of participants with depressive symptoms at both the WC and KZN sites scored in the mild depression range, with 9.5% and 2.9% in these ranges, respectively. Only 0.7% of participants from the WC site scored in the severe depression range, while none from the KZN site scored in this category. PHQ-9 scores distributions indicated that the WC site had participants across all PHQ-9 categories, while most participants from the KZN site scored within a mild range (Fig 1). The median PHQ-9 score was significantly higher for the WC site (4; IQR 2–5) compared to the KZN site (0; IQR 0–0) (p < 0.001). Most study participants from both locations received financial support from family and were high school students, though 1.8% of participants from the WC site reported having no formal education. The prevalence of sexually transmitted infections was high, with an overall prevalence of 56.8% (Table 1).

**Table 1:** Demographic characteristics of the study cohort - participants between the ages of 14 and 19 from the Western Cape and KwaZulu-Natal sites in South Africa.

Characteristics	WC site (N = 106) n(%)	KZN site (N = 168) n(%)	Total (N = 274) n(%)
**Age ranges**			
*18*–*19 years old*	70 (25.5)	84 (30.7)	154 (56.2)
*14*–*17 years old*	36 (13.1)	84 (30.7)	120 (43.8)
*Median age (Range)*	18 (16–19)	18 (14–19)	18 (14–19)
**Depressed**			
*Not depressed (0*–*4)*	72 (26.3)	159 (58.0)	231 (84.3)
*Mild (5*–*9)*	26 (9.5)	8 (2.9)	34 (12.4)
*Moderate (10*–*14)*	4 (1.5)	0 (0.0)	4 (1.5)
*Moderately severe (15*–*19)*	2 (0.7)	1 (0.4)	3 (1.1)
*Severe (20*–*27)*	2 (0.7)	0 (0.0)	2 (0.7)
**Level of education**			
*Tertiary*	12(4.4)	1 (0.4)	13 (4.7)
*High school*	88 (32.1)	164 (59,9)	252 (92.0)
*Primary school*	0 (0.0)	3 (1.1)	3 (1.1)
*No education*	5 (1.8)	0 (0.0)	5 (1.8)
*Missing*	*1*		
**Employment status**			
*Unemployed*	23 (8.4)	27 (9.9)	50 (18.2)
*Student/Learner*	80 (29.2)	141 (51.5)	221 (80.7)
*Formally employed*	1 (0.4)	0 (0.0)	1 (0.4)
*Other*	2 (0.7)	0 (0.0)	2 (0.7)
**Financial Support(Family support)** ^*^ ** **			
*Family*	104 (38.0)	153 (55.8)	257 (93.8)
*Partner*	0 (0.0)	1 (0.4)	1 (0.4)
*Social grant*	0 (0.0)	33 (12.0)	33 (12.0)
**Have you experienced death in the household**			
*Yes*	37 (13.5)	36 (13.1)	73 (26.6)
*No*	68 (24.8)	132 (48.2)	200 (73.0)
*Missing*	*1*		
**Bacterial vaginosis**			
*Absent*	39 (14.2)	58 (21.2)	97 (35.4)
*Present*	56 (20.4)	103 (37.6)	159 (58.0)
*Missing*	*11*	*18*	
**Sexually transmitted infections**			
*No STI*	37 (13.5)	93 (34.0)	130 (47.4)
*Neisseria gonorrhoeae*	16 (5.8)	14 (5.1)	30 (10.9)
*Chlamydia trachomatis*	53 (19.3)	55 (20.1)	108 (39.4)
*Trichomonas vaginalis*	8 (2.9)	10 (3.6)	18 (6.6)
*Positive for multiple STIs*	63 (23.0)	67 (24.5)	130 (47.4)
*Missing*	*11*	*8*	

* *some study participants reported more than one source of financial support.*

### Factors associated with depressive symptomology

In the univariate analysis (Table 2), younger adolescents (14–17 years) were less likely to exhibit depressive symptoms compared to older adolescents (Odds ratio (OR) 0.44; 95% confidence interval (CI) 0.22–0.90). Location was a strong predictor, with AGYW from the WC site having significantly higher odds of experiencing depressive symptoms (OR 8.34, 95% CI 3.80–18.30). Several risky sexual behaviours, including having more than four lifetime partners (OR 8.151; 95% CI 3.60–18.45), having a non-peer partner (OR 2.98, 95% CI 1.52–5.84), and engaging in sex under the influence of alcohol (OR 2.58; 95% CI 1.32–5.04) were all associated with increased odds of depressive symptomology.

**Table 2:** The association between socio-demographic, sexual history, HIV risk related behaviours, biological and substance use factors with prevalence of depressive symptomology in adolescent girls.

Variable	Depressive symptomology 43 (15.7%) n (row%)	No depressive symptomology 231(84.3%) n (row%)	Odds Ratio (OR) (95% CI)	Adjusted Odds Ratio (AOR) (95% CI)
**Age group**				
*14*–*17 years*	12(10.0)	108(90.0)	*0.44 (0.22–0.90)	0.80 (0.34–1.88)
*18*–*19 years(ref)*	31(20.1)	123(79.9)	1.0	1.0
**Location**				
*WC*	34(32.1)	72(67.9)	***8.34 (3.80–18.30)	***5.25 (1.95–14.17)
*KZN (ref)*	9(5.4)	159(94.6)	1.0	1.0
**Have you ever experienced death in the household?**				
*Yes*	16(21.9)	57(78.1)	1.80 (0.91–3.58)	
*No (ref)*	27(13.5)	173(86.5)	1.0	
*Missing*	0	1(100.0)		
**Family financial support**				
*Yes*	42 (16.3)	215 (83.7)	3.12 (0.40–24.17)	
*No (ref)*	1 (5.9)	16 (94.1)	1.0	
**Financial support**				
*Family(ref)*	41(17.6)	192 (82.4)	1.0	0.37 (0.05–3.01)
*Part/Social/Family*	1(2.8)	35(97.2)	*0.13 (0.02–1.01)	1.0
*missing*	1(20.0)	4(80.0)		
**Sexual debut age**				
*Below 16 yrs. (ref)*	11 (11.1)	88 (88.9)	1.0	
*16 yrs. and older*	32 (18.3)	143 (81.7)	1.79 (0.86–3.73)	
**Was the sexual debut consensual?**				
*Yes*	40(15.0)	227(85.0)	0.35 (0.03–3.98)	
*No (ref)*	1(33.3)	2(66.7)	1.0	
*Missing*	2(50.0)	2(50.0)		
**Number of lifetime partners**				
*One partner (ref)*	17 (9.4)	163 (90.6)	1.0	1.0
*2*–*3 partners*	9 (16.4)	46 (83.6)	1.88 (0.79–4.49)	1.12 (0.43–2.90)
*4 or more partners*	17 (46.0)	20 (54.0)	***8.15 (3.60–18.45)	***3.456 (1.24–9.60)
*Missing*	0	2 (100)		
**Have you ever had sex under influence of alcohol?**				
*Yes*	20(25.6)	58(74.4)	***2.58 (1.32–5.04)	1.06 (0.48–2.38)
*No (ref)*	23(11.8)	172(88.2)	1.0	1.0
*Missing*	0	2 (100)		
**Ever had a transactional sex?**				
*Yes*	3(27.3)	8(72.7)	2.08 (0.53–8.18)	
*No (ref)*	40(15.3)	222(84.7)	1.0	
*Missing*	0	1 (100)		
**Partner age gap**				
*Peers(ref* **)**	23(11.4)	178(88.6)	1.0	1.0
*Non peers*	20(27.8)	52(72.2)	**2.98 (1.52–5.84)	0.37 (0.05–3.01)
*Missing*	0	1(100)		
**Was the condom used in your last act?**				
*Yes*	21(25.6)	61(74. 4)	2.57 (1.32–5.0)	1.13 (0.49–2.62)
*No (ref)*	22(11.8)	164 (88.2)	1.0	1.0
*Missing*	0	6 (100)		
**Do you use sexual enhancer with your partner?**				
*Yes*	7(6.5)	100 (93.5)	***0.24 (0.10–0.57)	
*No (ref)*	36(22.5)	124 (77.5)	1.0	
*Missing*	0	7(100)		
**Ever been pregnant?**				
*Yes*	14(18.9)	60(81.1)	1.39 (0.69–2.82)	
*No (ref)*	28(14.4)	167 (85.6)	1.0	
*Missing*	1(20.0)	4(80.0)		
**Ever had miscarriage?**				
*Yes*	3 (60.0)	2 (40.0)	*7.42 (1.20–45.93)	
*No (ref)*	38 (16.8)	188 (83.2)	1.0	
*Missing*	2 (4.7)	41 (95.3)		
**Ever terminated pregnancy?**				
*Yes*	9 (42.9)	12 (57.1)	***5.13 (2.0–13.17)	
*No (ref)*	31 (12.8)	212 (87.2)	1.0	
*Missing*	3 (30.0)	7 (70.0)		
**Do you use contraceptive?**				
*Yes*	38 (19.4)	158 (80.6)	**3.46 (1.31–9.16)	
*No (ref)*	5 (6.5)	72 (93.5)	1.0	
*Missing*	0	1 (100)		
**Bacterial Vaginosis**				
*Yes*	23 (14.5)	136 (85.5)	0.86 (0.43–1.72)	
*No (ref)*	16 (16.5)	81 (83.5)	1.0	
*Missing*	4 (22.2)	14 (77.8)		
**Sexually Transmitted Infection**				
*Yes*	23 (17.7)	107 (82.3)	1.18 (0.61–2.28)	
*No (ref)*	20 (15.4)	110 (84.6)	1.0	
*Missing*		14 (100)		
**Do you use recreational drugs?**				
*Yes*	1 (11.1)	8 (88.9)	0.80 (0.10–6.61)	
*No (ref)*	34 (13.5)	218 (86.5)	1.0	
*Missing*	8 (61.5)	5 (38.5)		
**Do you consume alcohol?**				
*Yes*	19 (17.0)	93 (83.0)	1.70 (0.83–3.48)	
*No (ref)*	16 (10.7)	133 (89.3)	1.0	
*Missing*	8 (61.5)	5(38.5)		
**Body Mass Index**				
*Underweight-Normal (ref)*	25 (16.1)	130 (83.9)	1.0	
*Overweight*	17 (15.5)	93 (84.5)	0.95 (0.49–1.86)	
*Missing*	1 (11.1)	8 (88.9)		

The asterisk denotes the following: *p ≤ 0.05, **p ≤ 0.01, ***p ≤ 0.001.

Conversely, receiving financial support from more than one source (such as family, social grants, or partners) (OR 0.13; 95% CI 0.02–1.01) and the use of sexual enhancers (OR 0.24; 95% CI 0.10–0.57) were associated with lower odds of depressive symptomology, irrespective of location. Condom use was associated with higher odds of depressive symptoms in AGYW (OR 2.57; 95% CI 1.32–5.00). Additionally, experiences of miscarriage (OR 7.42; 95% CI 1.20–45.93), pregnancy termination (OR 5.13; 95% CI 2.00–13.17), and contraceptive use (OR 3.46; 95% CI 1.31–9.16) were all linked to increased odds of depressive symptoms.

**Fig 2:**
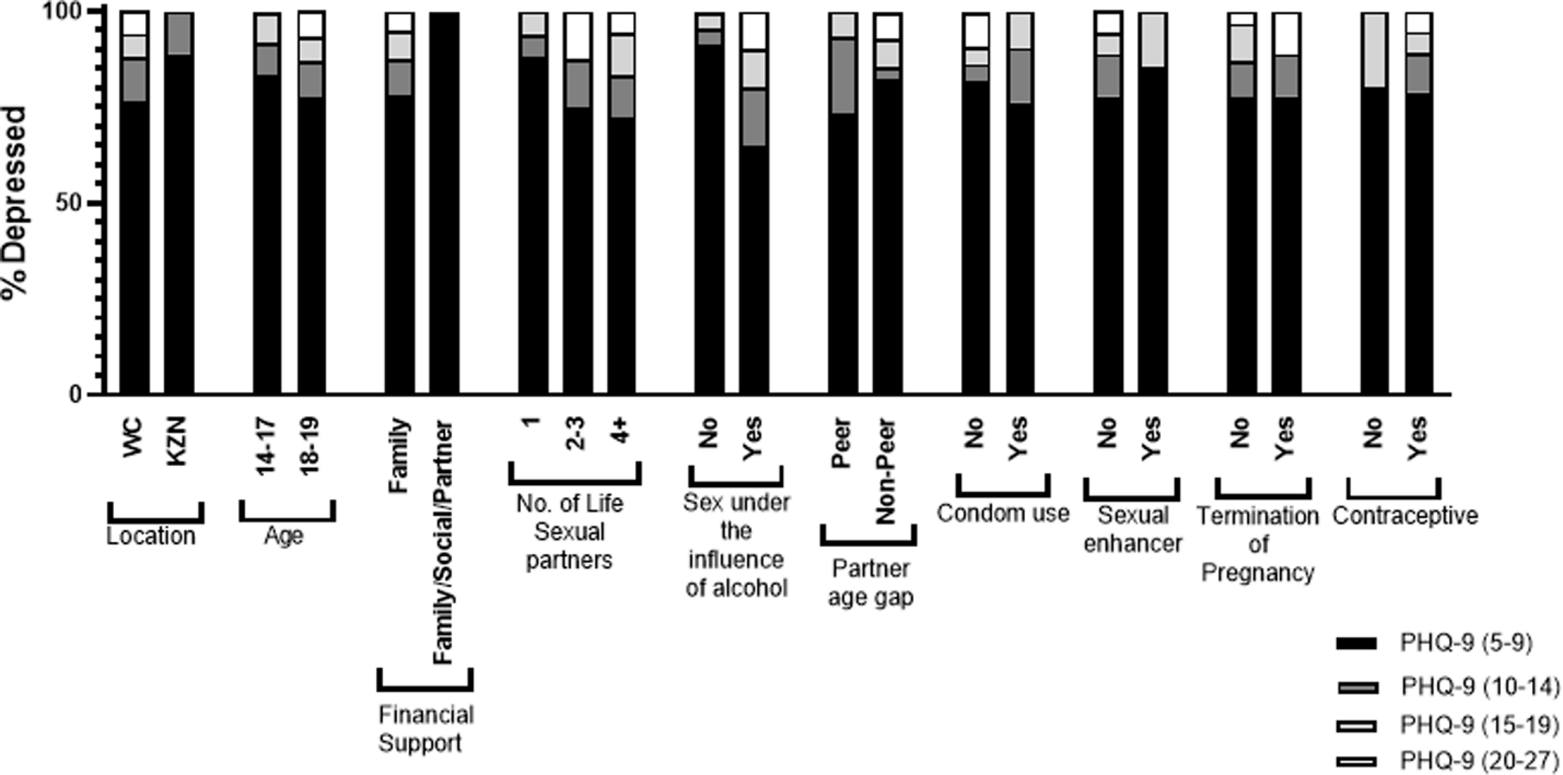
The distribution of depression categories; mild, moderate, moderately severe and severe across variables significantly associated with depression.

Variables that were significantly associated with depressive symptoms, in the univariate analysis, were further examined to identify which factors most contributed to the PHQ-9 depression categories (Fig 2). The PHQ-9 “Mild” category (scores of 5–9) was most prevalent across each significant variable. Variables such as having a higher number of lifetime sexual partners (2–3 partners), engaging in sex under the influence of alcohol, having a non-peer partner, not using a condom during intercourse, and terminating a pregnancy showed relatively high proportions within the “Severely Depressed”range (PHQ-9 scores of 20–27). The distribution of participants across each PHQ-9 category by site, as well as the socio-behavioural variables contributing to each level of depressive symptoms is shown in Fig 2.

The effects of age, location, number of lifetime partners, age difference with sexual partner, condom use, sex under the influence of alcohol and financial support were assessed using multivariate logistic regression (Table 2). The Hosmer-Lemeshow test results (Table 3) indicate that the model fits the observed data well, with a p-value of 0.17, suggesting no significant difference between observed and predicted values. Location was associated with more than a fivefold increase in the adjusted odds of experiencing depressive symptoms among participants in the study cohort (adjusted odds ratio [AOR] = 5.25; 95% CI 1.95–14.17). Additionally, having a higher number of lifetime partners (four or more) tripled the odds of depressive symptoms (AOR = 3.46; 95% CI 1.24–9.60).

**Table 3:** Hosmer-Lemeshow test.

Chi-square	df	p-value
11.547	8	0.173

## Discussion

Adolescent depression is an important public health issue that requires urgent and focused attention, especially in a setting as complex and diverse as South Africa. The prevalence of depressive symptomology obtained in this study (15.7%) is quite similar to what has been reported in the literature. Craig et al. suggest that the prevalence of depression in the South African adolescent population using the PHQ-9 ranges from 14.7% to 38.3% [21]. Age, location, multiple sexual partners, sex under the influence of alcohol, partner age gap, previous miscarriage, termination of pregnancy and contraceptive use were all significantly associated with increased odds of depressive symptomology in the univariate analysis. These are factors that, according to the literature, have been known to influence HIV risk in AGYW. However, these did not present as significant predictors in the multivariate analysis. Multivariate analysis found location and multiple sexual partners to be key influencers in the risk of presenting depressive symptoms.

Although the univariate data does not provide evidence of a cause, it is evident in research that multiple sexual partners, sex under the influence of alcohol and age disparate relationships have been associated with increased HIV acquisition risk [22–24]. The experience of a stressor such as the termination of an unexpected pregnancy results in a higher risk of depressive symptomology and binge drinking [25]. Our univariate findings suggest that these socio-behavioural factors are interconnected with depressive symptomology and HIV acquisition risk, and these interactions should be explored further.

The striking difference between the prevalence of depression between the two sites and the odds related to the WC site (WC site = 34% prevalence versus KZN site = 9% prevalence; 5 times greater odds of experiencing depressive symptomology when residing in WC) could be attributed to the differences in the characteristics of each community. The rural Vulindlela KZN site is a community characterised by traditional settlements or farmlands and an HIV prevalence of 36.3% [26,27], while the WC site in the area of peri-urban Philippi East is characterized by high unemployment rates, low per capita income, frequent instances of gangsterism and an HIV prevalence of 24%, the highest in the WC province [28–30]. Our study showed similar results to studies [31,32] where AGYW in urban settings were at greater risk of experiencing depression compared to those in more rural settings. Township settings often present increased exposure to various forms of interpersonal violence, crime, and socio-economic deprivation, that can negatively impact the mental and emotional wellbeing of those living in these areas [33]. Additionally, township settings often include people from multiple locations who may have less social coherence, and fewer formal support structures [34]. Rural settings often include households characterised by strong family connections, long established households, and a stronger sense of community with more collective support structures in place [34]. Structural differences between provinces may also mediate the risk of reporting depressive symptoms. A study by Herman et al [35], concurrent with our findings, showed that the WC province had the highest prevalence of mental health disorders when compared to other South African provinces. Geographical differences may be due to the Western Cape being one of the first regions to be colonised and is highly urbanised as compared to provinces with more rural areas such as KZN [35].

Secondly, our analysis shows that having multiple partners (4 or more) was strongly associated with increased odds (3.5 times likelihood) of being depressed. Considering that having multiple partners is also associated with increased HIV risk, this may indicate a point of synergy between depression and increased HIV risk. This result is similar to findings from other African settings, where reporting multiple partners; a known risky behaviour for HIV acquisition, was associated with increased odds of having depressive symptoms [36]. It is unclear whether depressive symptoms could increase the likelihood of AGYW engaging in risky sexual practices such as having multiple partners or rather that engaging in risky sexual behaviour may cause depression. This relationship needs to be explored in longitudinal research studies. Importantly, the higher odds of having multiple partners amongst AGYW showing depressive symptomology have important implications for their risk when we consider the high prevalence of other risk factors present in this sample. The majority of AGYW in our study reported not using a condom during sexual acts (69.4%), a high prevalence of non-peer relationships (26.4%), high rates of sex under the influence of alcohol (28.7%) and a prevalence of sexually transmitted infections (56.8%).

Collectively, the findings in this study highlight a possible synergy between depressive symptomology and HIV risk behaviours. If social factors (i.e., poverty, social dependence) enhance the risk of depressive symptomology and HIV, and depressive symptomology also increases the risk of HIV infection on its own, depression may serve to enhance the risk of both on its own and by affecting other pathways resulting in increased HIV risk.

There are some limitations to the current analysis. The data is cross-sectional and therefore we cannot establish causality. Future longitudinal studies are needed to investigate the causal pathways between the risk factors associated with HIV and depression. This study is nested under the ongoing parent study, and therefore the risk factors that were analysed were those limited to the data already collected from the parent study. Future exploration will require more in-depth and targeted data on all factors that could impact the risk of depression in the two different contexts. Nonetheless, this study highlights a need to establish more varied and nuanced risk profiles as all AGYW are not equally at risk of HIV or depression, however, those who present with one condition should also be screened for the other. Finally, future work should explore the rural site/urban divide within the same province, rather than different provinces.

## Conclusion

In conclusion, this research suggests an important connection between depression and HIV risk factors, highlighting the need for more comprehensive HIV prevention strategies. To effectively address the HIV epidemic, interventions must consider mental health screening and provide tailored psychosocial support, particularly for individuals living in urban and township areas. Furthermore, HIV risk assessments and prevention measures, including pre-exposure prophylaxis, should also incorporate mental health evaluations. By recognising and addressing these interconnected factors, we can work towards a more holistic approach to HIV prevention that may improve health outcomes and contribute to the resilience of communities.

## Supporting information

S1 Fig Consort diagram describing the cohort of adolescent girls included in the depressive symptomology sub-study. (TIF)
