# Supplementary figures and images for: Exploring the relationship between established HIV risk factors and depressive symptoms amongst young women without HIV in two sites in South Africa
Source: PLoS One. 2025 Jan 29;20(1):e0317732. doi: 10.1371/journal.pone.0317732 (PMC11778764; doi:10.1371/journal.pone.0317732)

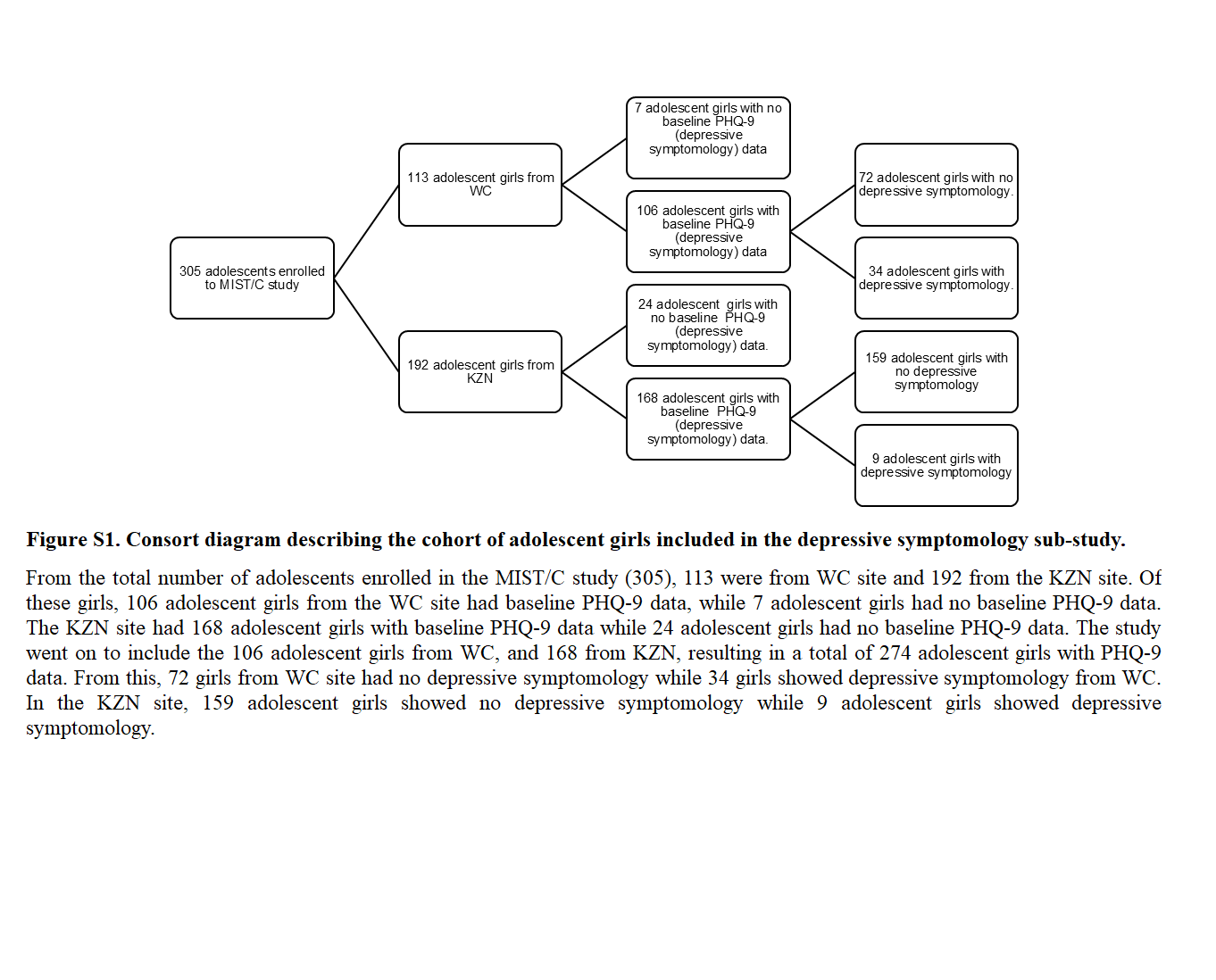

Supplement: S1 Fig — (TIF) [file pone.0317732.s001.tif]
